# Effects of intravenous butaphosphan and cyanocobalamin to late pregnant ewes on the metabolic indices around parturition and weight gain of their lambs after birth

**DOI:** 10.1002/vms3.687

**Published:** 2021-12-13

**Authors:** Azizollah Mohammadi Barimanloo, Aliasghar Chalmeh, Mehrdad Pourjafar, Abdolah Mirzaei

**Affiliations:** ^1^ Department of Clinical Sciences School of Veterinary Medicine Shiraz University Shiraz Iran

**Keywords:** butaphosphan, cyanocobalamin, metabolic disorders, parturition, pregnancy, sheep

## Abstract

**Background:**

Management and control of metabolic disorders in sheep around parturition is important. and various researchers have suggested different managerial solutions. Butaphosphan and cyanocobalamin are widely used for curing metabolic disorders resulting from poor nutrition, inadequate management or diseases.

**Objectives:**

It was hypothesised that butaphosphan and cyanocobalamin could improve the metabolism of ewes around parturition.

**Methods:**

Twenty‐eight clinically healthy 3‐year‐old pregnant Afshari ewes from 21 days before parturition were enrolled into four equal groups: control (Ctrl), B+C1, B+C2 and B+C3. The Ctrl group only received intravenous normal saline and B+C1, B+C2 and B+C3 ewes, respectively, received an intravenous combination of 10% butaphosphan and 0.005% cyanocobalamin at 2, 4 and 6 ml/ewe, on Days 19–21, 10–12 and 1–3 before parturition. Blood samples were taken from all the ewes on Days 21, 12 and 3 before lambing at parturition day and on days 3, 12 and 21 after parturition. A body condition score of all the ewes was assessed at blood sampling days, and lambs born from the ewes were weighed at birth and every 2 weeks up to 3 months. Serum concentrations of glucose, cortisol, non‐esterified fatty acids, beta‐hydroxy butyric acid, triglyceride, cholesterol, high‐, low‐ and very‐low‐density lipoproteins, aspartate aminotransferase and alanine aminotransferase were measured.

**Results:**

This drug combination decreased circulating glucose, cortisol, lipid profile and hepatic enzymes via dose‐dependent manner, 6 ml of this drug compound/ewe was more potent than 4 and 2 ml/ewe. The lambs’ weight from mothers receiving 6 ml of this combination was significantly higher than those of the others.

**Conclusions:**

It may be suggested that the intravenous administration of 6 ml/ewe of this combination for 3 consecutive days in three states before parturition had prophylactic effects on metabolic disorders of ewes and enhanced the lambs weight gain after birth.

## Graphical abstract

• Pregnancy and parturition induce metabolic alterations in sheep.

• Butaphosphan and cyanocobalamin could improve the metabolism of ewes following their administrations at the late pregnancy period.

• Butaphosphan and cyanocobalamin can be suggested as an effective combination in reducing metabolic disturbances in sheep.

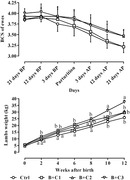

## 1 INTRODUCTION

Metabolic needs in sheep increase at the late pregnancy, making them susceptible to metabolic diseases, such as pregnancy toxaemia, which leads to different metabolic disturbances (Patton et al., 2007). This metabolic demand is mainly due to the synthesis of colostrum in the pre‐parturition period and milk production after lambing, causing metabolic changes in the haemostatic and adaptive processes that characterise this transition period. Fetal growth also increases the metabolic needs of pregnant ewes at the end of pregnancy. In addition, low dry matter intake raises negative energy balance and intensifies metabolic alterations (Castaneda‐Gutierrez et al., 2009). Colostrum production at the end of pregnancy and postpartum milk production further increase glucose demand with the mammary glands (Mattmiller et al., 2011).

The most important factor leading to metabolic disorders around parturition and particularly at the end of pregnancy in ewes is negative energy balance (Esposito et al., 2014). Pregnancy toxaemia as a common condition of ketonaemia in late pregnant ewes is the result of the excessive lipid mobilisation due to the negative energy balance and of the increased blood levels of non‐esterified fatty acids (NEFAs), which can be completely oxidised to carbon dioxide (CO_2_) and water (H_2_O) through Krebs cycle or partially oxidised to ketone bodies, such as beta‐hydroxybutyric acids (BHBA). This situation also affects the body condition of ewes and strategies as the control of body condition is used to minimise the negative energy balance in ruminants (Xue et al., 2019).

Therefore, researchers have claimed that providing energy to ewes during this period can be effective in reducing metabolic deficiencies (Chibiza et al., 2008); hence, glucose supply is necessary for efficient metabolism to counteract the negative energy balance, but glucose metabolism in ruminants is different from that of the other mammals. Moreover, glucose in ruminants usually has to be produced by hepatic gluconeogenesis from glucose precursors (Chalmeh et al., 2015).

The combined use of butaphosphan, as an organic source of phosphorus, and cyanocobalamin‐promoting gluconeogenesis affects milk production (Kreipe et al., 2011). A negative energy balance of dairy cows has also been suggested for ewes (Pereira et al., 2013). Cyanocobalamin is a type of Vitamin B_12_ shown to decrease in cows around calving (Kincaid & Socha, 2007). It has been hypothesised that Vitamin B_12_ injections may increase gluconeogenesis by increasing the activity of methylmalonyl‐CoA mutase, a Vitamin B_12_‐dependent enzyme in the tricarboxylic acid cycle (Kennedy et al., 1990). As a source of organic phosphorus, butaphosphan may lead to gluconeogenesis since the intermediates in gluconeogenesis must be phosphorylated to continue the cycle (Berg et al., 2006). This compound can directly interfere with certain metabolic pathways, but it is assumed that it still has an indirect effect on hepatic metabolism, mainly in the processes of oxidation and ketogenesis. Improvements in the energy status of animals treated with these substances have been reported (Rollin et al., 2010). In addition, previous studies have revealed that this combination can be effective in reducing the occurrence of negative energy balance and insulin resistance in dairy cows (Chalmeh et al., 2021). However, it is not yet clear whether this form of phosphorus is biologically available to animals (Rollin et al., 2010).

Several researchers also evaluated the effects of this compound on some metabolic characteristics of ewes at pre and post‐partum periods (Pereira et al., 2013; Temizel et al., 2015). Concerning previous studies on the role of butaphosphan and cyanocobalamin in promoting metabolic levels in ruminants, the present work investigates the metabolic effects of intravenous injection of butaphosphan and cyanocobalamin at three different doses into pregnant ewes on 3 consecutive days in three stages before lambing to evaluate their metabolic indices around parturition and their lambs’ weight after birth. Regarding the importance of managing the metabolism of ewes around parturition, we considered a management method to improve their metabolic levels. Therefore, in this study, the interactions of butaphosphan and cyanocobalamin on ewes’ metabolism around lambing were investigated. The results of the current research may aid to better manage the metabolism of ewes around parturition.

## 2 MATERIALS AND METHODS

### 2.1 Animals

The present experimental study was conducted after being approved by the Iranian laboratory animal ethics framework under the supervision of the Iranian Society for the Prevention of Cruelty to Animals and Shiraz University Research Council (IACUC no: 4687/63). Twenty‐eight clinically healthy 3‐year‐old pregnant Afshari ewes (50 ± 5 kg body weight) with a history of at least one successful parturition were randomly selected from a farm in Bojnourd (37.4702° N, 57.3143° E), the capital city of North Khorasan Province, Iran. Afshari sheep are among the Iranian fat‐tailed breed and have a high growth rate. This breed has thick wool fibers. The good production of meat and high‐fat milk of this sheep has made it one of the dual‐purpose meat–dairy sheep. It has high adaptability to the climatic conditions of cold regions. All the ewes in this study were kept in open‐shed barns with free access to water and shade. The ewes used in this study were fed manually before and during the study and did not have free grazing in the pasture. Ingredients and chemical composition of pre‐ and post‐partum diets are presented in Table 1. Their pregnancy was confirmed based on a history of mating, artificial insemination and ultrasonographic examinations, and the approximate time of parturition was estimated. All ewes were entered into the study and grouped based on the above‐mentioned parameters. In addition, the occurrence of pre‐parturition clinical signs (such as enlargement and oedema of the mammary glands, the appearance of fatty secretions at the tip of the nipple and their filling, loosening of the ligaments at the base of the tail, swelling and loosening of the vulva) were carefully evaluated. The ewes were included in the study at 21 days before lambing and were randomly divided into four equal groups, namely, control (Ctrl), B+C1, B+C2 and B+C3. All ewes lambed naturally and unassisted as nature intended. They gave birth to their lambs in lambing paddocks in order to minimise disturbances of lambing ewes. Good observation was performed at lambing time. There was not any intervention during lambing of studied sheep.

**TABLE 1:** Ingredients and chemical composition of pre‐ and postpartum diets (DM basis)

**Items**	**Periods**	
	**Pre‐parturition**	**Post‐parturition**
Ingredients (gram)		
Alfalfa hay	540	765
Corn silage	690	1035
Wheat straw	540	540
Corn	180	315
Barley	270	405
Soybean meal	90	180
Vitamin‐mineral premix	20	20
Salt	10	10
Limestone	10	10
Chemical composition		
DMI (g/day)	2350	3280
CP (% of DMI)	10	13
TDN (% of DMI)	60	70
ME (Mcal/kg)	2.16	2.52

Abbreviations: CP, crude protein; DM, dry matter; DMI, dry matter intake; ME, metabolisable energy; TDN, total digestible nutrients.

It is worth mentioning that this study initially commenced on 36 ewes, and eight of them were excluded from the study due to inconsistency of parturition date with drug injection days. The injection times in ewes that remained in this study were completely in agreement with the time of their parturition.

### 2.2 Experimental procedures

The Ctrl group only received intravenous normal saline and the B+C1, B+C2 and B+C3 ewes received an intravenous combination of 10% butaphosphan and 0.005% cyanocobalamin (Catosin 10%, Erfan Pharmaceutical Company) at 2, 4 and 6 ml/ewe, respectively. All the ewes were grouped 21 days prior to lambing and were sampled up to 21 days following parturition. Normal saline in the Ctrl group and butaphosphan and cyanocobalamin combination in the B+C groups were administrated on Days 19–21, 10–12 and 1–3 before parturition. Blood samples were taken from all the ewes on days 21, 12 and 3 before lambing at parturition day and 3, 12 and 21 days after parturition. Body condition score (BCS) of all the ewes was assessed, at blood sampling days, based on the visual and tactile assessment of body fat reserves employing a 5‐point scale with a 0.25‐point increase (Kenyon et al., 2014). In this scoring system, a score of 1 indicates severe thinness, whereas a score of 5 implies overweightness. The lambs born from the ewes were weighed at birth and every 2 weeks up to 3 months.

### 2.3 Serological and biochemical assays

The samples were collected in 10‐ml plain tubes and centrifuged within 2 h after collection at 3000 × g for 10 min at room temperature. Sera were stored at –22°C until assayed. Glucose was examined with an enzymatic (glucose oxidase) colorimetric method (ZistChem; sensitivity equal to 5 mg/dl; intra‐assay and inter‐assay CV < 10% and 11%, respectively). NEFA and BHBA were assayed via the colorimetric method (Randox; sensitivity for NEFA and BHBA equal to 0.072 and 0.100 mmol/L, respectively; intra‐assay and inter‐assay CV for NEFA < 7% and 11% and for BHBA < 5% and 6%, respectively). Cortisol was assayed via competitive immunoenzymatic colorimetric method (Diametra; sensitivity equal to 2.42 ng/ml; intra‐assay and inter‐assay CV ≤ 5.1% and 11%, respectively). Triglyceride (TG; sensitivity equal to 3 mg/dl), cholesterol (sensitivity equal to 5 mg/dl), high‐density lipoprotein (HDL; sensitivity equal to 1 mg/dl), aspartate aminotransferase (AST; sensitivity equal to 4 U/L) and alanine aminotransferase (ALT; sensitivity equal to 3 U/L) were measured by the use of produced biochemical kits by Biorexfars Company. Low‐density lipoprotein (LDL; sensitivity equal to 0.3 mg/dl) was measured with a commercial kit manufactured by Man Company. Values of very LDL (VLDL) were obtained by dividing TG values to 5 (Friedewald et al., 1972).

### 2.4 Statistical analyses

Data are expressed as mean ± standard error (SE). A linear mixed model for repeated measures was applied to study the effects of treatment, sampling days before and after parturition and their interaction on the measured variable. In each mixed model, the measured values of different variables were considered as the dependent variable. The groups of treatment and sampling days of peri‐parturient periods were utilised as the fixed effects and individual ewes as random effects. We employed Bonferroni as the post hoc test to find the differences among the groups and determine estimated means from the repeated measures linear mixed model. Statistical analyses were performed using the SPSS software (SPSS for Windows, version 22, SPSS Inc), and the mean difference was considered significant at the *P* < 0.05 level.

## 3 RESULTS

**FIGURE 1:**
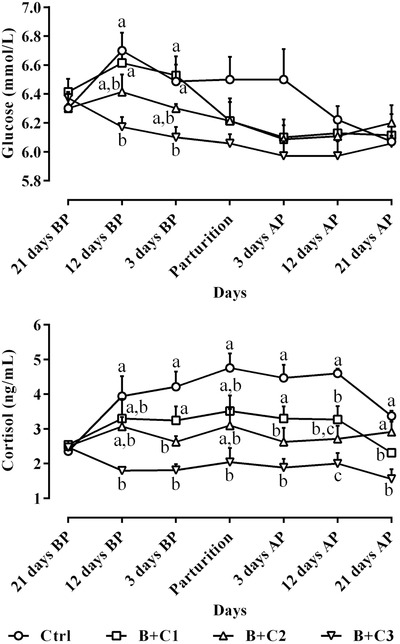
Alterations of circulating glucose and cortisol levels (mean ± standard error (SE)) from 21 days before to 21 days after parturition in ewes following intravenous administration of 10% butaphosphan and 0.005% cyanocobalamin at 2, 4 and 6 ml/ewe on Days 19–21, 10–12 and 1–3 before parturition and comparison of these parameters among different studied groups during the study period. AP, after parturition; B+C1, B+C2 and B+C3, butaphosphan + cyanocobalamin at 2, 4 and 6 ml/ewe, respectively; BP, before parturition. ^a,b,c^ Different letters indicate significant differences among studied groups on similar days (*P* < 0.05)

**FIGURE 2:**
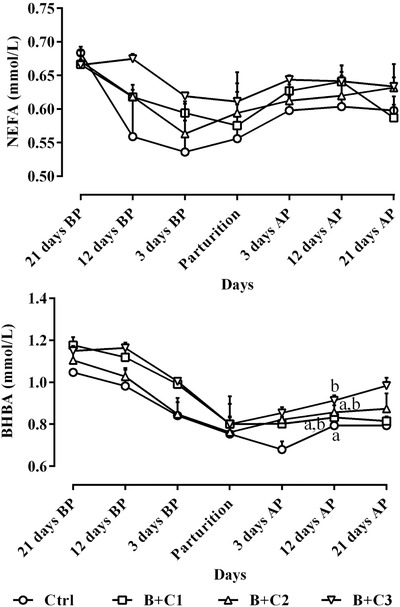
Alterations of circulating NEFA and BHBA levels (mean ± SE) from 21 days before to 21 days after parturition in ewes following intravenous administration of 10% butaphosphan and 0.005% cyanocobalamin at 2, 4 and 6 ml/ewe on Days 19–21, 10–12 and 1–3 before parturition; and comparison of these parameters among different studied groups during the study period. AP, after parturition; B+C1, B+C2 and B+C3, butaphosphan + cyanocobalamin at 2, 4 and 6 ml/ewe, respectively; BHBA, beta‐hydroxybutyric acid; BP, before parturition; NEFA, non‐esterified fatty acid. ^a,b^ Different letters indicate significant differences among studied groups at similar days (*P* < 0.05)

**FIGURE 3:**
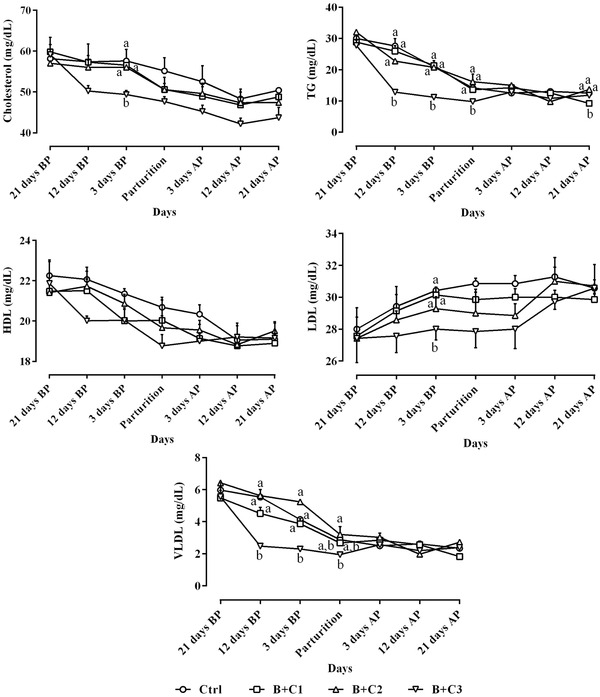
Alterations of circulating TG, cholesterol, HDL, LDL and VLDL levels (mean ± SE) from 21 days before to 21 days after parturition in ewes following intravenous administration of 10% butaphosphan and 0.005% cyanocobalamin at 2, 4 and 6 ml/ewe on Days 19–21, 10–12 and 1–3 before parturition and comparison of these parameters among different studied groups during the study period. AP, after parturition; B+C1, B+C2 and B+C3, butaphosphan + cyanocobalamin at 2, 4 and 6 ml/ewe, respectively; BP, before parturition; HDL, LDL and VLDL, high‐, low‐ and very low‐density lipoprotein; TG, triglyceride. ^a,b^ Different letters indicate significant differences among studied groups at similar days (*P* < 0.05)

**FIGURE 4:**
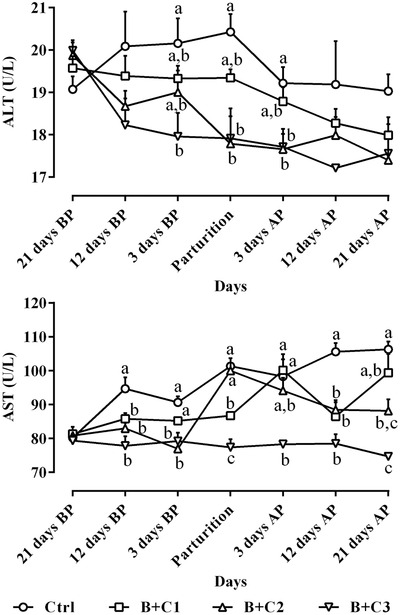
Alterations of circulating AST and ALT levels (mean ± SE) from 21 days before to 21 days after parturition in ewes following intravenous administration of 10% butaphosphan and 0.005% cyanocobalamin at 2, 4 and 6 ml/ewe on Days 19–21, 10–12 and 1–3 before parturition and comparison of these parameters among different studied groups during the study period. ALT, alanine aminotransferase; AP, after parturition; AST, aspartate aminotransferase; B+C1, B+C2 and B+C3, butaphosphan + cyanocobalamin at 2, 4 and 6 ml/ewe, respectively; BP, before parturition. ^a,b,c^ Different letters indicate significant differences among studied groups on similar days (*P* < 0.05)

**FIGURE 5:**
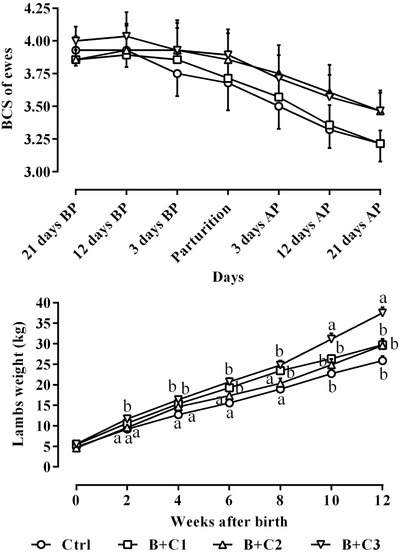
Alterations of BCS of ewes (mean ± SE) from 21 days before to 21 days after parturition in ewes following intravenous administration of 10% butaphosphan and 0.005% cyanocobalamin at 2, 4 and 6 ml/ewe on Days 19–21, 10–12 and 1–3 before parturition; and comparison of the BCS among different studied groups during the study period. Alterations of weight gain of the lambs after birth and comparison of their weight among studied groups. AP, after parturition; B+C1, B+C2 and B+C3, butaphosphan + cyanocobalamin at 2, 4 and 6 ml/ewe, respectively; BCS, body condition score; BP, before parturition. ^a,b^ Different letters indicate significant differences among studied groups at similar days (*P* < 0.05)

The alterations of the studied parameters are presented in Figures 1, 2, 3, 4, 5. Table 2 shows the effect of treatment groups on each parameter, their changes during the study and the interaction of different study groups during the pre‐ to postpartum periods.

**TABLE 2:** *P*‐value of treatment, sampling times and their interaction effects in linear mixed models analysis for each parameter

**Parameters**	***P*‐value**		
	**Treatment**	**Time**	**Treatment × time**
Glucose	0.014*	< 0.001*	0.100
Cortisol	< 0.001*	< 0.001*	0.003*
NEFA	0.032*	< 0.001*	0.984
BHBA	0.004*	< 0.001*	0.894
TG	< 0.001*	< 0.001*	< 0.001*
Cholesterol	0.065	< 0.001*	0.854
HDL	0.242	< 0.001*	0.766
LDL	0.094	< 0.001*	0.957
VLDL	< 0.001*	<0.001*	< 0.001*
AST	< 0.001*	< 0.001*	< 0.001*
ALT	0.001*	< 0.001*	0.162
BCS	0.799	< 0.001*	0.954
Lambs weight	<0.001*	<0.001*	< 0.001*

Abbreviations: ALT, alanine aminotransferase; AST, aspartate aminotransferase; BCS, body condition score; BHBA, beta‐hydroxy butyric acid; HDL, high‐density lipoprotein; LDL, low‐density lipoprotein; NEFA, non‐esterified fatty acid; TG, triglyceride; VLDL, very low‐density lipoprotein.

* indicates significant effects (*P* < 0.05).

Figure 1 demonstrates the peri‐parturient alterations of circulating glucose and cortisol levels following intravenous butaphosphan and cyanocobalamin administration to the late pregnant ewes. Glucose decreased from pre‐ to post‐parturition periods in all the studied ewes. The lowest concentrations of glucose were detected in B+C3 group. These differences were significant in 12 and 3 days before parturition (Figure 1; *P *< 0.05). Cortisol increased and decreased significantly during the study period in Ctrl and B+C3 groups, respectively. The significant lowest concentrations of cortisol were detected in the B+C3 group following butaphosphan and cyanocobalamin administration from 21 days before to 21 days after parturition (Figure 1; *P* < 0.05).

Figure 2 reveals the alterations of circulating NEFA and BHBA levels in this research. There were no significant differences among all the studied ewes (Figure 2; *P* > 0.05) and the interactions of treatment and time about NEFA and BHBA during the study period were also non‐significant (Table 2; *P* > 0.05).

Alterations of the lipid profile are presented in Figure 3. The decreasing trend changes of TG, cholesterol, HDL and VLDL, and increasing alterations of LDL were detected from 21 days before to 21 days after parturition during the study period. The lowest concentrations of TG, cholesterol, LDL and VLDL were detected in the B+C3 group, and these differences were of significance in some days and not in all the studied days (Figure 3).

Changes in circulating ALT and AST levels are presented in Figure 4. The results of the present study demonstrated that the significant lowest concentrations of both enzymes were detected in the B+C3 group (*P* < 0.05). Ctrl ewes had the highest levels of AST and ALT during the study period significantly (Figure 4; *P* < 0.05).

Changes in BCS of the studied ewes are presented in Figure 5. Based on Figure 5 and Table 2, all the ewes had a significant decreasing pattern of BCS during the study period (*P* < 0.05), but there were no significant differences among the studied ewes (*P *> 0.05). Alterations of lambs’ weight from birth to 12 weeks later are depicted in Figure 5. The results reveal the significant increasing weight for all the studied lambs (Table 2; *P* < 0.05), and the significant highest weights were detected in lambs born from the B+C3 mothers, and the significant lowest weight were in the Ctrl group (*P* < 0.05). All the sheep in this study gave birth to a lamb and none were pregnant with twins.

## 4 DISCUSSION

Improving the metabolic state of ewes during the peri‐parturient period has an important role to manage and control their metabolic problems. We hypothesised that the intravenous administration of butaphosphan and cyanocobalamin combination to late pregnant ewes may influence their metabolic performance from pre‐ to post‐parturition periods. The results of the current research showed that this drug combination reduced circulating glucose, cortisol, lipid profile and hepatic enzymes in a dose‐dependent manner, 6 ml of this drug compound/ewe was more potent than 4 and 2 ml/ewe, respectively. Furthermore, the weight of the lambs from mothers receiving 6 ml of this combination was significantly higher than those of the others.

In the present work, the serum cortisol concentration in the B+C3 group was significantly lower than that of the other groups (Figure 1; *P* < 0.05), and the cause of this effect is still unclear; meanwhile, what has already been proven is the role of cortisol in metabolic disorders, such as pregnancy toxaemia in the ewes around parturition (Hosseini et al., 2018). Therefore, a significant decrease in cortisol in the groups receiving butaphosphan and cyanocobalamin and specifically the B+C3 group indicated the proper efficacy of this compound in reducing metabolic disorders in ewes. On the other hand, cortisol has an effective role in gluconeogenesis in ruminants and lower glucose concentration in the B+C3 group, compared to other groups, possibly due to lower cortisol levels in this group (Figure 1). According to the results, on one hand, the difference in glucose levels between different groups on most sampling days was not significant, and on the other hand, lower glucose levels in the B+C3 group did not put them in a hypoglycemic state.

The results of Pereira et al. (2013) implied that glucose levels are significantly increased in treated animals, which is consistent with the mechanism of action of cyanocobalamin, used as a co‐factor for methylmalonyl CoA mutase and the entry of fatty acids into the Krebs cycle as a key in gluconeogenesis (Kennedy et al., 1990). Cyanocobalamin is produced in the gastrointestinal tract of ruminants, but its level in the blood decreases immediately after parturition (Girard & Matte, 1999). This is because before parturition, most fetal growth occurs and nutrients are delivered to produce colostrum (Duehlmeier et al., 2011). Thus, the administration of cyanocobalamin supplements provides the body with the energy it needs by inducing gluconogenic function (Brozos et al., 2011). Depending on the energy available to the animal, the body may select gluconeogenesis or initiate the mechanism of lipolysis simultaneously to supply energy to the peripheral tissues (Kalapos et al., 1996). In addition, animal hepatocytes oxidise large amounts of fatty acids due to lack of energy and raise the amount of nicotinamide adenine dinucleotide (NADH) in mitochondria (Enjalbert et al., 2001).

Previous studies on the effects of butaphosphan and cyanocobalamin on cortisol changes in sheep and cattle have not stated significant changes in this hormone although some have shown that this combination reduces cortisol release in heifers (Hansel et al., 1992), the mechanism of which is unknown. Some researchers have also reviewed the insulin levels in animals receiving butaphosphan and cyanocobalamin. Herein, insulin was not measured, but the assessment of insulin resistance via conventional methods can help better understand the function of this drug and its effects on metabolism. In a study by Chalmeh et al. (2021), insulin resistance was lower in cows receiving higher doses of butaphosphan and cyanocobalamin than in other treatments and Ctrl groups and a similar study could be conducted to better evaluate the metabolic effects of butaphosphan and cyanocobalamin in sheep. In general, sheep at the end of pregnancy, especially those with more than one embryo, experience a negative energy balance, and their bodies utilise stored fat to compensate. A major problem during this period is the decreased responsiveness and insulin sensitivity; however, glucose requirements increase two to three times (Overton & Waldron, 2004). Tabele (2017) examined the combination of butaphosphan and cyanocobalamin on postpartum glucose metabolism in dairy cows. The compound was injected intramuscularly on Days 7, 12, 17, 22 and 27 postpartum. On Days 8 and 28 postpartum, the cows underwent glucose tolerance and insulin challenge tests. They reported that the groups did not differ in glucose metabolism on Days 8 and 28 (*P* > 0.05). They also concluded that the combined application of butaphosphan and cyanocobalamin had a positive effect on the adaptation of glucose metabolism in dairy cows in the early lactation period.

Vitamin B_12_ is an effective vitamin for the metabolism of carbohydrates, lipids and proteins. Vitamin B_12_ is synthesised by rumen microorganisms in the presence of cobalt in ruminants. Diet composition is also an important factor in the synthesis of Vitamin B_12_ by microorganisms (Grace & Knowles, 2012). Vitamin B_12_ also affects the function of enzymes affecting lipid metabolism, which may be why Vitamin B_12_ deficiency may alter lipid metabolism. In studies by Stangl et al. (1999) and Kumar et al. (2013), increased cholesterol was observed in cows and rats lacking cobalamin, but in the study of Furll et al. (2010) on the effects of butaphosphan and cyanocobalamin on dairy cows, no differences were found between cholesterol levels among groups. The results of the current study showed that butaphosphan and cyanocobalamin did not have a significant effect on changes in lipid profile among the Ctrl and treatment groups, and the differences observed between these groups were mostly not significant. Only TG and VLDL levels in the B+C3 group were significantly lower in the pre‐partum periods than those in the other groups. Due to the fact that no significant differences were observed between the lipomobilisation indices which are NEFA and BHBA between the groups and the effects of butaphosphan and cyanocobalamin on glucose metabolism were not significant, no significant differences were consequently observed between the components of the lipid profile in this research.

Fat mobilisation is a major feature of the transition period in dairy cows and a similar period in ewes. Since the energy they need is not provided by the diet, they consume more energy than they receive and, therefore, have to use up their energy reserves. Intravenous injections of butaphosphan and cyanocobalamin into late‐lactating dairy cows have been revealed to reduce postpartum fat mobilisation as a side effect of increasing the use of fatty acids and reducing energy demand. The available data highlight possible functional mechanisms related to butaphosphan and cyanocobalamin, such as severe reduction in dry matter intake, improved gluconeogenesis and near‐parturition lipolysis and reduced depth and duration of negative energy balance in early lactation (Rollin et al., 2010). Recent studies have shown that butaphosphan and cyanocobalamin increase the expression of liver X receptor‐α mRNA, a key messenger in lipid metabolism, and stimulate the citric acid cycle (Nuber et al., 2016).

In this study, liver health was assessed with AST and ALT assessments. The results showed that the levels of these two enzymes in the B+C3 group were significantly lower than those of the other Ctrl and treatment groups, demonstrating the role of butaphosphan and cyanocobalamin in the health and function of hepatocytes. Liver health plays an important role in preventing metabolic disorders in ruminants, and other researchers have evaluated the effects of this compound on liver health indicators, such as total bilirubin (Antunes et al., 2019; Şahal et al., 2017; Temizel et al., 2015), AST (Pereira et al., 2013; Tabele, 2017) and gamma‐glutamyl transferase (Pereira et al., 2013). Therefore, the use of butaphosphan and cyanocobalamin plays an effective role in reducing the incidence of metabolic disorders by maintaining and promoting liver health and function. It is well known that the liver of dairy cows undergoes significant adaptations upon initiation of lactation, and as a result, most dairy cows experience certain degrees of hepatic lipidosis (Hayirli, 2006). Improving the overall health of the liver and its ability to process energy can have potential effects on negative energy balance in early lactation (De Koster & Opsomer, 2013). As a compound of organic phosphorus, butaphosphan is seriously involved in hepatic carbohydrate metabolism, in which all the mediators in the gluconeogenic pathway must be phosphorylated. Hence, the rate of gluconeogenesis and glycolysis is regulated through the availability of phosphorus (Berg et al., 2006). Girard et al. (2005) hypothesised that lactogenesis failure to respond to folic acid supplements could be due to insufficient cyanocobalamin supply.

The deficiency of cyanocobalamin causes the accumulation of methylmalonyl‐CoA, which in turn prevents the beta‐oxidation of fatty acids in the liver and can lead to the accumulation of triacylglycerol. Cows receiving cyanocobalamin supplementation are likely to reduce the accumulation of methylmalonyl‐CoA and allow beta‐oxidation of fatty acids to continue, and as long as the source of oxaloacetate is sufficient, acetyl‐CoA molecules enter the Krebs cycle to provide energy (Graulet et al., 2007). Kennedy et al. (1990) stated that methylmalonyl‐CoA plays a unique regulatory role in gluconeogenesis and beta‐oxidation of fatty acids. In fact, methylmalonyl‐CoA inhibits carnitine palmitoyltransferase I, which converts long‐chain fatty acids to their carnitine esters allowing them to enter the mitochondria in which beta‐oxidation takes place. When gluconeogenesis and the glucose propionate‐Krebs cycle pathway are active, the concentration of methylmalonyl‐CoA increases and inhibits beta‐oxidation (Longo et al., 2016). However, the concentration of methylmalonyl‐CoA and then methylmalonic acid increases once the entry of propionate into the Krebs cycle is slowed with cyanocobalamin deficiency. Consequently, beta‐oxidation is reduced although gluconeogenesis is also reduced (Takahashi‐Iñiguez et al., 2012). Concentrations of NEFA and BHBA in this study, as the main indicators of negative energy balance and lipomobilisation in ruminants, indicated that butaphosphan and cyanocobalamin had no effect on their changes and many researchers found no statistically significant difference between Ctrl and treatment groups of sheep (Pereira et al., 2013; Temizel et al., 2015), and our findings in the current work confirm their results.

Phosphorus is an effective element for carbohydrate metabolism, gluconeogenesis and adenosine triphosphate (ATP) production (Hers, 1990). In a study by Rollin et al. (2010) in dairy cows, no differences in phosphorus levels were observed between the groups receiving butaphosphan and cyanocobalamin, but Furll et al. (2010) found a non‐significant increase in phosphorus levels. Temizel et al. (2015) investigated the prophylactic effects of butaphosphan and cyanocobalamin on pregnancy toxaemia in sheep and found no difference in phosphorus levels between the study groups, compared with the Ctrl ones. This may be on account of the short half‐life of phosphorus and the fact that butaphosphan is a source of organic phosphorus (EMEA, 1999).

Temizel et al. (2015) found that BHBA levels in the ewes receiving butaphosphan and cyanocobalamin in the third week before lambing were significantly lower than those in the Ctrl group but did not observe a statistically significant difference between the groups in the postpartum weeks. Meanwhile, the lambs in the treatment group weighed more than the lambs born in the Ctrl one. They stated that these results indicate that the combination of butaphosphan and cyanocobalamin was partially effective in preventing subclinical pregnancy toxaemia in the last weeks of pregnancy.

Rollin et al. (2010) administered butaphosphan and cyanocobalamin to 510 dairy cows and observed a significant decrease in BHBA concentration. In another study, Furll et al. (2010) found that BHBA and NEFA levels decreased following intravenous injection of butaphosphan and cyanocobalamin. Pereira et al. (2013) observed an increase in dry matter intake and a decrease in the serum levels of NEFA and BHBA after the administration of butaphosphan and cyanocobalamin to ewes on lambing day and on the following second and fourth days although these differences were not of statistical significance. They mentioned that the combination of butaphosphan and cyanocobalamin regulates energy status and improves the appetite of ewes after parturition. Herein, the levels of BHBA and NEFA decreased in the groups treated with butaphosphan and cyanocobalamin, yet there were no statistically significant differences between the studied groups, possibly due to the amount of drug prescribed. The findings of Temizel et al. (2015) implied that administration of higher doses of this compound can be effective in preventing pregnancy toxaemia in ewes. Pereira et al. (2013) found that the administration of butaphosphan and cyanocobalamin to pregnant ewes increased dry matter intake and decreased BHBA levels in the first week after parturition, which was associated with decreased lipomobilisation and ketogenesis. These results are similar to previous observations in dairy cows (Rollin et al., 2010; Pereira et al., 2013). Gordon et al. (2017) evaluated the combined effects of butaphosphan and cyanocobalamin on the improvement of ketosis and milk production of dairy cows in early lactation and reported that butaphosphan and cyanocobalamin could be useful in the treatment of ketosis in dairy cows. In another study on ewes, the effect of concomitant injection of butaphosphan and cyanocobalamin on the prevention of pregnancy toxaemia in the pre‐parturition period was investigated (Tamizel et al., 2015). They concluded that the combination of butaphosphan and cyanocobalamin could be an alternative treatment to prevent pregnancy toxaemia. Rollin et al. (2010) investigated the effect of injection of butaphosphan and cyanocobalamin on the day of parturition and the next day on the prevalence of subclinical ketosis in dairy cows in the early postpartum period. According to their results, this injection could reduce the prevalence of subclinical ketosis during the week after calving in adult dairy cows. Pereira et al. (2013) investigated the effect of butaphosphan and cyanocobalamin supplementation on plasma metabolites in dairy cows in the postpartum period. All the cows were injected every 5 days from calving to 20 days postpartum. In their presented results, increasing doses of butaphosphan and cyanocobalamin caused a linear decrease in plasma concentrations of NEFA and BHBA. In another study, cows with subclinical ketosis in early lactation were used to examine the effects of butaphosphan alone or in combination with cyanocobalamin on their metabolism. Their findings suggested that injection of cyanocobalamin with butaphosphan until early lactation in dairy cows with ketosis affected lipid metabolism by affecting plasma metabolites, which are likely to be affected by the modification of key factor activity in the liver.

Changes in the ewes’ physiological condition from the dry and pregnant to the non‐pregnant and lactating stages are the most critical period that they expose to most metabolic, productive and reproductive disorders. The increase in their metabolic needs during this period is accompanied by an increase in the need for micronutrients, amino acids and energy, in which inadequate supply of these nutrients and metabolites makes animals more susceptible to a range of metabolic and infectious diseases. Therefore, nutrients and micronutrients play an important role in the pre‐to postpartum periods (Keshri et al., 2021). There is evidence that micronutrients are involved in functions such as the intracellular detoxification of free radicals, the synthesis of reproductive steroids and other hormones, carbohydrates and proteins and nucleic acid metabolism. The deficiency of micronutrients may also impair fertility, fetal growth and survival, postpartum recovery activities, milk production and offspring growth and survival (Smith & Akinbamijo, 2000). Hence, supplementation by micronutrients positively affects metabolic performances of ewes around parturition. The results of the current research also revealed that parenteral administration of 10% butaphosphan and 0.005% cyanocobalamin compound at 6 ml/ewe at pre‐partum period improved their metabolism around parturition and their lamb weight gain after birth.

Weight loss and reduced body scores of ewes during lambing and subsequent periods are normal findings, revealing that ewes are in a negative energy balance following lactogenesis. However, this decrease in body score occurred in all the studied ewes, and no statistically significant difference was observed among the different groups. On the other hand, the weight gain of lambs whose mothers were in the B+C3 group was significantly higher than that of the other lambs, thereby confirming the results of previous studies (Tamizel et al., 2015). This finding may be due to the promotion of hepatic, lipid metabolism and the reduction of cortisol levels, which may have controlled and reduced the negative energy balance in the B+C3 ewes. Based on the results of this research, the metabolic performances of ewes receiving 6 ml of this compound were significantly improved in comparison with other groups. It may be suggested that managing negative energy balance and lipomobilisation of ewes by administration of 6 ml of this compound may positively affect the lamb's weight gain. The weight of lambs at birth was close to each other in the different groups, and no statistically significant differences were observed among the groups (Figure 5; *P* > 0.05). However, with the increasing age of lambs, the weight of lambs born from the mothers of the B+C3 group was significantly higher than the lambs of other mothers (*P *< 0.05). One of the probable reasons for this finding may be due to the effect of butaphosphan and cyanocobalamin combination on milk composition. However, in this research, we did not study milk production and its compounds. Another possible reason for this finding may be the effect of this drug compound on the metabolism of lambs after birth.

## 5 CONCLUSION

Metabolic profiles of the ewes were changed around parturition and butaphosphan and cyanocobalamin administration at pre‐parturition period could alter their metabolic profile at pre‐ and post‐partum periods. It may be suggested that intravenous administration of 6 ml/ewe of this combination on 3 consecutive days at three states before parturition had prophylactic effects on metabolic disorders of ewes and enhanced the weight gain of the lambs after birth.

## ETHICS STATEMENT

The present experimental study was conducted after being approved by the Iranian laboratory animal ethics framework under the supervision of the Iranian Society for the Prevention of Cruelty to Animals and Shiraz University Research Council (IACUC no: 4687/63).

## CONFLICT OF INTEREST

The authors certify that there is no conflict of interest.

## AUTHOR CONTRIBUTION

*Investigation, methodology, project administration, resources, writing–original draft*: Azizollah Mohammadi Barimanloo. *Data curation, formal analysis, methodology*: Abdolah Mirzaei. Aliasghar Chalmeh: Conceptualization, Methodology, Formal analysis, Investigation, Resources, Data curation, Writing original draft, Writing – review & editing, Supervision, Project administration. Mehrdad Pourjafar: Validation, Resources, Writing – review & editing, Supervision, Project administration.
